# Comparison of clinical and laboratory profile of pulmonary and extrapulmonary tuberculosis in children: A single-center experience from India

**Published:** 2021-07-16

**Authors:** Sachin Singh, Madhuradhar Chegondi, Swathi Chacham, Prawin Kumar, Jagdish Prasad Goyal

**Affiliations:** ^1^Department of Pediatrics, Post Graduate Institute of Medical Education and Research, Chandigarh, India; ^2^Department of Pediatrics, Division of Pediatric Critical Care Medicine, Stead Family Children’s Hospital, Carver College of Medicine, University of Iowa, Iowa, United States; ^3^Department of Pediatrics, All India Institute of Medical Sciences, Rishikesh, Uttarakhand, India; ^4^Department of Pediatrics, All India Institute of Medical Sciences, Jodhpur, Rajasthan, India

**Keywords:** tuberculosis, pulmonary, extrapulmonary, children, clinical, microbiological, India

## Abstract

**Background:**

Pediatric tuberculosis (TB) is an indicator of the recent transmission of TB in the community. However, the diagnosis of pediatric TB poses a challenge to clinicians.

**Aims:**

We aimed to evaluate and compare the clinical and laboratory profile of pulmonary TB (PTB) and extra PTB (EPTB) in children and adolescents.

**Methods:**

In this retrospective observational study, children attending the pediatric TB clinic of All India Institute of Medical Sciences, Rishikesh, from August 2015 to July 2017 were included in the study. The medical case records of patients were reviewed for demography, clinical findings, investigations, and diagnosis. The clinical and laboratory characteristics of patients with PTB and EPTB were compared.

**Results:**

A total of 58 children included. Out of which, 33 (56.9%) had PTB, and 25 (43.1%) had EPTB. The EPTB cases included 15 (60%) pleural TB, 9 (36%) lymph node TB, and 1 (4%) TB meningitis patient. Fever, cough, and weight loss were the most common symptoms. Hilar lymphadenopathy was the most common radiological abnormality. Microbiological confirmation was possible in 54.5% of patients with PTB. Cough (aOR 70.326; 95% CI: 5.370–921.032) and microbiological confirmation (aOR 46.011; 95% CI: 2.073–1021.201) were more in PTB as compared to EPTB.

**Conclusions:**

PTB and EPTB are common in children and adolescents. The typical clinical manifestations and positive microbiological confirmation are less common in EPTB than PTB.

**Relevance for patients:**

TB is one of the common communicable diseases in the developing world. Diagnosis of TB in children is often challenging. Our study results help in better understanding childhood TB and EPTB clinical features and have potential to increase diagnostic yield.

## 1. Introduction

According to the World Health Organization (WHO) report, around 10 million people get affected by tuberculosis (TB) every year [1]. The global burden of TB cases shows a significant variation geographically from <5 cases to more than 500 new cases per 100,000. Of them, 44% of cases were from Southeast Asia, and India alone accounts for 27% of the total global TB burden. TB affects all age groups irrespective of gender, with the highest incidence among males aged >15 years. Children aged <15 years account for 11% of total TB cases, with an equal incidence between male and female children [2]. In India, pediatric TB cases accounted for 6% of the total TB burden due to underdiagnosis. In comparison, the actual pediatric burden is closer to 8%. In 2018, a total of 132,711 pediatric TB patients (only 59% of estimated) were notified in India, which included new and relapsed pediatric TB [3]. TB typically affects lung (pulmonary TB [PTB]), but it can also affect other sites (extra PTB [EPTB]) [4].

Pediatric TB is difficult to diagnose due to its paucibacillary nature and lower chance of bacteriological confirmation [5]. Pediatric TB is an indicator of the recent transmission of TB in the community [6]. Given the lower yield of bacterial confirmation in children, establishing the pediatric TB diagnosis is challenging in developing countries like India due to limited resources [7]. Studies from Asia, Middle East, Europe, and the USA have reported epidemiology and clinical manifestation of pediatric TB [8-11]. Clinical presentation of pediatric TB varies among regions due to epidemiological situation and HIV burden in the host country [12]. Furthermore, the diagnosis of pediatric TB also varies depending on available resources in the countries [13].

Moreover, there is a lack of data from different parts of India regarding pediatric TB epidemiological and clinical profile. Therefore, this study was conducted to determine the clinical profile and compare PTB and EPTB in pediatric TB patients.

## 2. Methods

### 2.1. Study setting

This retrospective, observational study was carried out at a tertiary care center, All India Institute of Medical Sciences (AIIMS) in Rishikesh, Uttarakhand, India, in the Pediatric Department. Our institutional review board approved the study. AIIMS, Rishikesh, serves patients from the urban and rural areas of Uttarakhand and Western Uttar Pradesh, located in India’s northern part. The pediatric TB clinic was started at our institute in August 2015. Guidelines of the revised national TB program (RNTCP) are followed to diagnose TB in all pediatric patients [14]. The treatment of all TB patients was also done as per RNTCP guidelines.

#### 2.1.1 Data collection

All children aged <18 years visiting pediatric TB outpatient clinic from August 2015 to July 2017 were included in the study. We have used our pediatric TB clinic structured format to collect the study variables. Both microbiologically confirmed cases and clinically diagnosed cases were included in the study. Microbiologically confirmed cases were defined as those with (a) positive sputum or gastric aspirate or other body secretions such as pleural fluid, cerebrospinal fluid, or surgical biopsy smear for acid-fast bacilli or (b) detection of *Mycobacterium* TB in cartridge-based nucleic acid amplification test or (c) TB culture. Clinically diagnosed cases were defined based on symptoms, suggestive radiology, and positive tuberculin skin test (TST) results. Definition of symptoms was adapted from the RNTCP 2015 guidelines: Persistent fever and/or cough for more than 2 weeks with the loss of weight/no weight gain and/or history of contact with infectious TB case [15].

The medical record was reviewed for clinical, radiological, microbiological, and laboratory details. Besides demographic and anthropometric data, we also collected data about TST results, history of close contact with an active TB case, prior Bacille Calmette-Guerin (BCG) vaccination, and/or presence of BCG scar (at least four millimeters in size). The TST was performed by injecting 0.1 mL of 2 TU of purified protein derivative (PPD) intradermally into the middle one-third of the volar surface of the left forearm. The skin induration size was measured after 72 h [14]. TST considered positive if the induration size was more than 10 mm. For our analysis, we classified the data as PTB and EPTB groups.

### 2.2. Statistical analysis

Data analysis was performed using STATA 13.0 (STATA Corp., College Station, TX). Categorical data were expressed as counts and percentages, while continuous data were expressed as median and interquartile ranges (IQR). Differences between categorical data were analyzed using the Chi-square (χ^2^) or Fisher’s exact tests when cell sizes were <5. The Mann–Whitney U-test analyzed differences between continuous data. All statistical tests were two tailed, and *P*<0.05 was considered statistically significant. Multivariate logistic regression analysis was applied to adjust the confounding variables.

## 3. Results

A total of 58 children were analyzed. Out of them, 56.9% (*n*=33) had PTB and 43.1% (*n*=25) had EPTB. Among the EPTB cases, 60% (*n*=15) were pleural, 36% (*n*=9) lymph nodal, and 4% (*n*=1) with TB meningitis. None of them had disseminated TB. The majority of the children were female, 74.1% (*n*=43), and the median age was 14 (11–16) years. The median body mass index (BMI) of included children was 14.9 (14–16.4) kg/m^2^.

Fever was present in 93.9% of PTB and 76% of EPTB patients, while cough was present in 90.9% and 24% of PTB and EPTB patients, respectively. Weight loss and loss of appetite were present in 84.9% and 93.9% of PTB patients, whereas those were present in 66.7% and 60% of EPTB patients. Lymph node involvement was present in 24% of EPTB. History of contact with TB was present in 24% and 40% of patients with PTB and EPTB. Around two-thirds of both pulmonary and EPTB received the BCG vaccine. The BMI was not significantly different between the PTB and EPTB children (Table 1).

**Table 1:** Comparison of clinical profile of pulmonary and EPTB

Variables	PTB (*n*=33)	EPTB (*n*=25)	*P*-value
Median age in years (IQR)	15 (14–16)	13 (10–15)	0.01
Gender, *n* (%)			
Female	25 (75.8)	18 (72)	0.75
Median BMI (kg/m^2^) (IQR)	14.6 (13.8–16.1)	15.4 (14.2–17.2)	0.18
Area of residence, *n* (%)			
Urban	23 (69.7)	18 (72)	0.85
Rural	10 (30.3)	7 (28)	
Fever, *n* (%)	31 (93.9)	19 (76)	0.06
Cough, *n* (%)	30 (90.9)	6 (24)	<0.001*
Weight loss, *n* (%)	28 (84.9)	16 (66.7)	0.1
Loss of appetite, *n* (%)	31 (93.9)	15 (60)	<0.002*
Lymph node enlargement, *n* (%)	0 (0.0)	6 (24)	NR
Contact history, *n* (%)	8 (24.2)	10 (40)	0.19
BCG vaccinated, *n* (%)	23 (69.7)	17 (68)	0.64
BCG scar, *n* (%)	21 (63.6)	11 (44)	0.15

NR: Not reported; BCG: Bacille Calmette-Guerin;

* *P*-value<0.05, PTB: Pulmonary tuberculosis, EPTB: Extrapulmonary tuberculosis

The median hemoglobin and erythrocyte sedimentation rates were not significantly different between PTB and EPTB groups, while the median total leukocyte count was high in PTB. Abnormal chest radiograph was found in all children with PTB and only 22.4% of EPTB. Microbiological confirmation of TB was possible in 54% of patients with PTB and only in 4% of EPTB patients. Laboratory details are summarized in Table 2.

**Table 2:** Comparison of laboratory profile of pulmonary and EPTB

Variables	PTB (*n*=33)	EPTB (*n*=25)	*P*-value
Median Hb (gm/dL) (IQR)	10.7 (9.4–11.2)	10.4 (9.3–11.3)	0.792
Median total leukocyte count (×10^9^/L) (IQR)	11350 (10050–16200)	8700 (6500–10300)	0.001*
Median ESR (mm/1^st^ h) (IQR)	10.7 (9.4–11.2)	10.4 (9.3–11.3)	0.792
TST (>10 mm) *n* (%)	25 (75.7)	23 (92)	0.12
Abnormal chest X-ray, n (%)	33 (100)	3 (52)	NR
Lymphadenopathy	20 (60.6%)		
Cavity lesion	8 (24.2%)		
Miliary TB	5 (15.2%)		
Microbiologically confirmed TB, *n* (%)	18 (54.5)	1 (4)	<0.01*

NR: Not reported, IQR: Interquartile range; ESR: Erythrocyte sediment rate; TST: Tuberculin skin test;

* P-value <0.05, PTB: Pulmonary tuberculosis, EPTB: Extrapulmonary tuberculosis

On bivariate analysis of clinical and laboratory parameters, older age, cough, positive microbiological confirmation, and high total leukocyte counts were significantly associated with PTB compared to EPTB (*P*<0.05). On multivariate analysis, cough (aOR 70.326; 95% CI: 5.370–921.032) and positive microbiological confirmation (aOR 46.011; 95% CI: 2.073–1021.201) were significantly associated with PTB (Table 3).

**Table 3:** Multivariate analysis of clinical and laboratory parameters of pulmonary and EPTB

Variable	aOR	(95% CI)	*P*-value
Age	1.072	(0.819–1.402)	0.615
Cough	70.326	(5.370–921.032)	0.001*
Loss of appetite	1.127	(0.066–19.235)	0.934
Microbiological confirmation	46.011	(2.073–1021.201)	0.015*

Dependent variable: Pulmonary TB, aOR: Adjusted odds ratio; CI: Confidence interval, PTB: Pulmonary tuberculosis, EPTB: Extrapulmonary tuberculosis

All patients received isoniazid, rifampicin, pyrazinamide, and ethambutol for 2 months in the intensive phase, while isoniazid, rifampicin, and ethambutol for 4 months in the continuation phase. However, one patient with TB meningitis received the continuation phase for 10 months as per the RNTCP guideline. More than 90% of patients were compliant with the treatment. The most common cause of non-compliance was gastrointestinal upset. There was no mortality among study participants. However, we found post-treatment sequelae in around 50% of patients in the form of fibrosis, loss of lung volume, and pleural thickening.

## 4. Discussion

This retrospective observational study compared the clinical and laboratory profile of children with PTB and EPTB. We found that cough and microbiological confirmation of TB were more frequent in PTB than EPTB patients. Pleural TB was the most common form of EPTB followed by lymph node TB. In other studies evaluating pediatric TB, pleural TB was also the most common form of EPTB [16]. A study from Turkey reported lymph node TB as the most common form of EPTB in children [17].

Children with EPTB were younger in our study. Younger age has been reported in children with EPTB in the previous studies [8,18]. EPTB has low recovery, higher mortality, and relapse rate as compared to PTB in children. It may be the reason that younger children are more susceptible to EPTB [19]. In our study, there was female preponderance. A similar finding was also observed in children and adolescents with drug-resistant TB from another study from India [20]. In India, especially in rural areas, gender inequality still exists in many social aspects, including health-care access. Our cohort’s predominant female gender might be explained by the lack of early medical attention seeking by the parents and tertiary care center referral when severely ill. However, our cohort does not represent the community gender distribution for childhood TB.

Fever, cough, and weight loss were frequent clinical features in children with PTB, similar to other studies [21-24]. A refined symptom-based approach to diagnose PTB in children also suggested that cough, weight loss, and fatigue had good diagnostic accuracy in children [25]. The TB program in India also suggested screening any child for TB who had a fever and/or cough for more than 2 weeks along with weight loss and family history of contact with TB [26].

Contact history is essential for diagnosing TB in children since most of the children acquire TB from adults. The incidence of contact history varies between 16% and 40% in different studies [27-29]. In our study, the contact history was present in 26% and 40% of children with PTB and EPTB, respectively.

BCG vaccine is effective against TB meningitis and the dissemination of TB [30]. In India, the BCG vaccine is provided free of cost under the national immunization program. However, only two-thirds of our patients received the BCG vaccine. The current literature suggests that the BCG vaccine does not prevent primary infection, latent, or reactivation TB. No significant difference in the incidence of pediatric TB reported with and without BCG vaccination [31].

In this study, TST was positive in 75% of patients with PTB and 92% of patients with EPTB. It does not differ significantly between PTB and EPTB. TST-positive rate was similar in our study as compared to another study [32]. In India, TST is used for screening TB. TST positive indicates that the patient has a TB infection, not the disease. TST is limited by several factors such as the technique of administration, previous BCG vaccination, and nutritional status of patients [33]. Although the history of BCG vaccination may cause false-positive TST, the working group on TB, Indian Academy of Pediatrics recommended TST positive if the induration is 10 mm or more irrespective of BCG status. In addition, existing literature suggests a minimal effect on PPD reaction with the prior BCG vaccination [15,34].

The median hemoglobin level was slightly low in the present study and TLC was significantly elevated in PTB compared to EPTB. However, ESR was mildly elevated in both PTB and EPTB. A study from India to assess hematological parameters in TB also showed anemia and leukocytosis [35]. ESR is a non-specific test and depends on several factors. In a study from Qatar, authors reported normal ESR (<10 mm/h) in one-third of children and elevated ESR (≥10 mm/h) in two-third of children at the time of diagnosis [36]. The level of Hb, TLC, and another marker of inflammation varies in TB and may not be considered diagnostic for any form of TB.

The radiological findings in pediatric TB are variable. The suggestive radiological findings in children with TB include hilar lymphadenopathy, miliary TB, and fibrocavitary lesions [37]. Abnormal chest X-ray was present in all patients of PTB. The most common X-ray finding was hilar adenopathy. Other authors also reported a similar observation [38]. The radiological abnormalities were also detected in half of the patients with EPTB.

Microbiological confirmation is the gold standard for the diagnosis of TB. It also helps identify drug-resistant TB [39]. Microbiological confirmation is not always possible in children due to the paucibacillary nature of pediatric TB and difficulty in acquiring the sample. However, we were able to make microbiological confirmation by GeneXpert in 54% of PTB patients compared to 4% of patients of EPTB. Only one PTB patient was found to be rifampicin resistant in our study; the culture and sensitivity detected resistance to both isoniazid and rifampicin. A similar microbiological confirmation rate was reported by another study [40].

The study’s strength is that it is one of the few studies from India that compared clinical and laboratory profiles of PTB and EPTB children. The limitations of our study include the small sample size and retrospective nature. There were some missing data due to incomplete medical records. Moreover, our study was single center and not adequately powered to identify risk factors for PTB and EPTB.

## 5. Conclusions

PTB and EPTB are common in children and adolescents. The most common clinical presentation in EPTB was fever and weight loss. Cough and positive microbiological confirmation are significantly lower in EPTB compared to PTB. Common EPTB sites were pleura and lymph nodes. Microbiological diagnosis is rarely possible in EPTB. Therefore, the clinician should keep a high index of suspicion for EPTB so that the treatment of EPTB would not be delayed.

### Conflict of Interest

The authors declare no conflicts of interest.
